# The Stomatognathic System as an Expanding Frontier for Botulinum Toxin Therapy

**DOI:** 10.3390/toxins18100428

**Published:** 2026-10-02

**Authors:** Kazuya Yoshida

**Affiliations:** Department of Oral and Maxillofacial Surgery, National Hospital Organization, Kyoto Medical Center, 1-1 Mukaihata-cho, Fukakusa, Fushimi-ku, Kyoto 612-8555, Japan; omdystonia@gmail.com; Tel.: +81-75-641-9161; Fax: +81-75-643-4325

**Keywords:** botulinum toxin, stomatognathic system, dentistry, oral and maxillofacial surgery, oromandibular dystonia, bruxism, temporomandibular disorder, trigeminal neuralgia, sialorrhea, masseter prominence

## Abstract

Botulinum neurotoxin (BoNT) therapy has expanded from focal chemodenervation to a versatile therapeutic modality used across multiple medical specialties. The stomatognathic system is particularly suited to BoNT therapy because motor, sensory, articular, secretory, and aesthetic functions converge within a compact anatomical region. Current and emerging applications include oromandibular dystonia, dyskinesia, bruxism, tremor, hemimasticatory spasm, embouchure dystonia, masticatory muscle and temporomandibular disorder-related pain, trigeminal neuralgia, recurrent temporomandibular joint dislocation, sialorrhea, Frey syndrome, masseter prominence, gummy smile, and perioral wrinkles. Evidence and regulatory status vary substantially among indications, and many applications remain off-label. Dentistry and oral and maxillofacial surgery have historically played a limited role in BoNT therapy despite their detailed anatomical and functional expertise in this region. However, interest is increasing, and the recent approval of onabotulinumtoxinA in Japan for masseter prominence in adults younger than 65 years represents an important milestone. This review synthesizes current applications, limitations, safety considerations, and future directions, and proposes the stomatognathic system as a major expanding frontier for BoNT therapy.

## 1. Introduction

Botulinum neurotoxin (BoNT), produced by Clostridium botulinum, has evolved from a focal chemodenervating agent into a therapeutic platform with neuromuscular, autonomic, and antinociceptive applications.

BoNT produces temporary, reversible chemodenervation by inhibiting acetylcholine release at cholinergic nerve terminals through cleavage of soluble N-ethylmaleimide-sensitive factor attachment protein receptor (SNARE) proteins [1,2,3,4,5,6]. Beyond neuromuscular blockade, BoNT can modulate nociceptive mediator release and peripheral sensitization, providing a mechanistic basis for selected analgesic applications [7,8].

At the molecular level, BoNTs are composed of a heavy chain that mediates selective binding and endocytosis at cholinergic nerve terminals and a zinc-dependent light-chain protease that cleaves SNARE proteins required for synaptic vesicle fusion. After receptor binding, internalization, and translocation of the light chain into the cytosol, BoNT/A cleaves SNAP-25 and thereby suppresses acetylcholine release. The resulting functional denervation is temporary because neuromuscular transmission gradually recovers through synaptic remodeling and restoration of exocytotic machinery [2,3,4,5,6]. The same presynaptic mechanism underlies inhibition of cholinergic autonomic transmission in salivary and sweat glands, whereas analgesic effects may additionally involve reduced peripheral release of nociceptive mediators and modulation of peripheral sensitization [7,8]. These distinct biological actions provide the mechanistic basis for the unusually broad stomatognathic applications discussed in this review.

Since its initial therapeutic use for strabismus, BoNT has been adopted across neurology, ophthalmology, otolaryngology, rehabilitation medicine, dermatology, urology, pain medicine, gastroenterology, and aesthetic medicine [1,9]. One anatomical and functional region with particularly broad therapeutic potential, however, has received comparatively limited attention: the stomatognathic system.

The stomatognathic system is an integrated anatomical and functional unit comprising the maxilla and mandible, teeth, dental arches, temporomandibular joints (TMJs), masticatory muscles, tongue, lips, cheeks, palate, suprahyoid muscles, salivary glands, and associated neural and vascular structures. These structures contribute to mastication, swallowing, speech, respiration, facial expression, and oral competence. Unlike many regions dominated by a single physiological function, the stomatognathic system combines motor, sensory, articular, nociceptive, secretory, and aesthetic functions within a compact and anatomically complex space.

This functional diversity makes the stomatognathic system suitable for several pharmacological actions of BoNT. Reduction of excessive muscle activity may improve dystonia, spasm, bruxism, or joint instability; modulation of peripheral nociceptive signaling may contribute to pain relief; inhibition of cholinergic autonomic transmission reduces glandular secretion; and controlled weakening or volume reduction of selected muscles can modify facial morphology for aesthetic purposes.

Potential indications therefore extend well beyond a single disease category. They include oromandibular dystonia (OMD), oral dyskinesia, bruxism, tremor, hemimasticatory spasm, functional movement disorders, embouchure dystonia, temporomandibular disorder (TMD)-related muscle pain, other chronic orofacial pain conditions, trigeminal neuralgia, recurrent TMJ dislocation, sialorrhea, Frey syndrome, masseter prominence, gummy smile, and facial and perioral wrinkles [10,11,12,13,14,15,16,17,18,19,20,21,22].

Many of these conditions are common or chronic and can substantially impair eating, speech, appearance, and social functioning. Yet, awareness of BoNT therapy among dental professionals remains uneven. Disorders caused by abnormal motor control may instead prompt repeated treatment directed at the teeth, occlusion, prostheses, or TMJs. Conversely, BoNT used without an adequate diagnosis may result in unnecessary treatment, excessive muscle weakness, impaired mastication, dysphagia, facial asymmetry, or other complications.

Accordingly, expansion of BoNT therapy into dentistry requires more than wider access to injection. It requires integration of neurological diagnosis, dental and maxillofacial anatomy, functional assessment, injection guidance, dose optimization, and appropriate patient selection.

A previous review focused on BoNT therapy for movement disorders of the stomatognathic system [10]. The present review takes a broader perspective by conceptualizing the stomatognathic system as a multidimensional therapeutic field encompassing movement disorders, pain, TMJ dysfunction, salivary and autonomic disorders, and aesthetic applications. We examine why this region may represent an important expanding frontier for BoNT therapy and identify the scientific, clinical, regulatory, and educational challenges that must be addressed for safe development.

## 2. Materials and Methods

### 2.1. Review Approach

This narrative, clinically oriented review synthesized evidence relevant to current and emerging applications of BoNT therapy in the stomatognathic system. Evidence was interpreted hierarchically, with priority given to systematic reviews and meta-analyses, consensus or standard-of-care documents, randomized controlled trials, representative case series for rare disorders, and regulatory documents when relevant. Because the available evidence varies substantially in study design, quality, clinical maturity, and regulatory status across indications, the clinical position categories used in Table 1 are descriptive and should not be interpreted as formal GRADE recommendations.

For this narrative review, targeted literature searches were performed in PubMed/MEDLINE through 27 September 2026, supplemented by reference-chain searching of key reviews and consensus documents and by regulatory documents from relevant authorities. Search terms were combined as appropriate and included “botulinum toxin” or “botulinum neurotoxin” with “oromandibular dystonia”, “lingual dystonia”, “bruxism”, “temporomandibular disorder”, “orofacial pain”, “trigeminal neuralgia”, “temporomandibular joint dislocation”, “sialorrhea”, “Frey syndrome”, “masseter prominence”, “gummy smile”, “occlusal trauma”, and “periodontitis”. English-language systematic reviews, meta-analyses, consensus statements, randomized trials, and major regulatory documents were prioritized. For uncommon disorders for which controlled studies are sparse, representative case series and specialist reports were included. This was not a systematic review; no formal PRISMA flow or risk-of-bias meta-analysis was undertaken.

### 2.2. Use of Generative Artificial Intelligence

**Figure 1:**
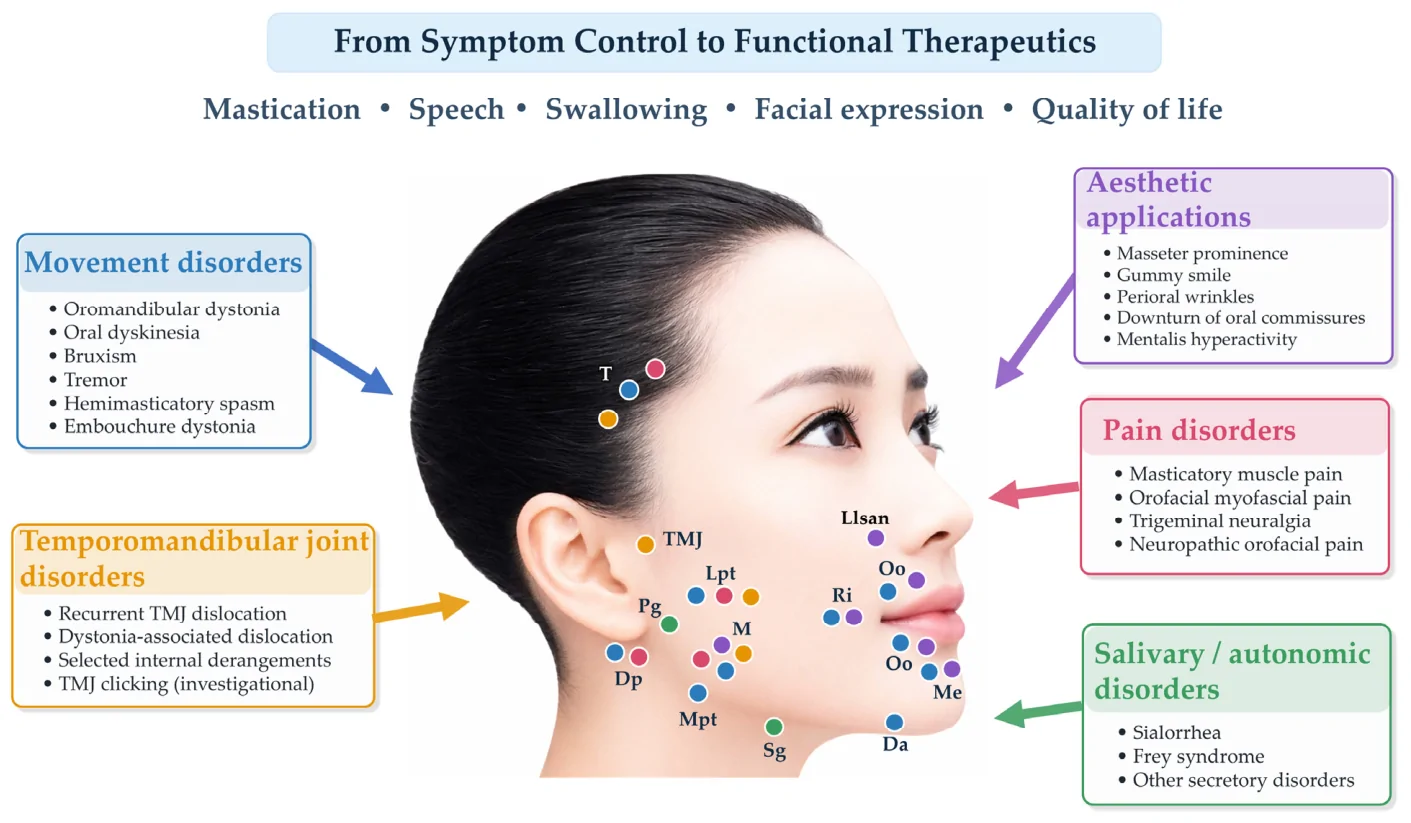
The stomatognathic system as an expanding frontier for BoNT therapy. The stomatognathic system integrates motor, sensory, articular, autonomic, and aesthetic functions, enabling broad applications of BoNT therapy across movement disorders, pain disorders, temporomandibular joint disorders, salivary/autonomic disorders, and aesthetic indications. T, temporalis; M, masseter; TMJ, temporomandibular joint; Pg, parotid gland; Sg, submandibular gland; Lpt, lateral pterygoid; Mpt, medial pterygoid; Llsan, levator labii superioris alaeque nasi; Ri, risorius; Oo, orbicularis oris; Me, mentalis; Da, anterior digastric; Dp, posterior digastric.

**Figure 2:**
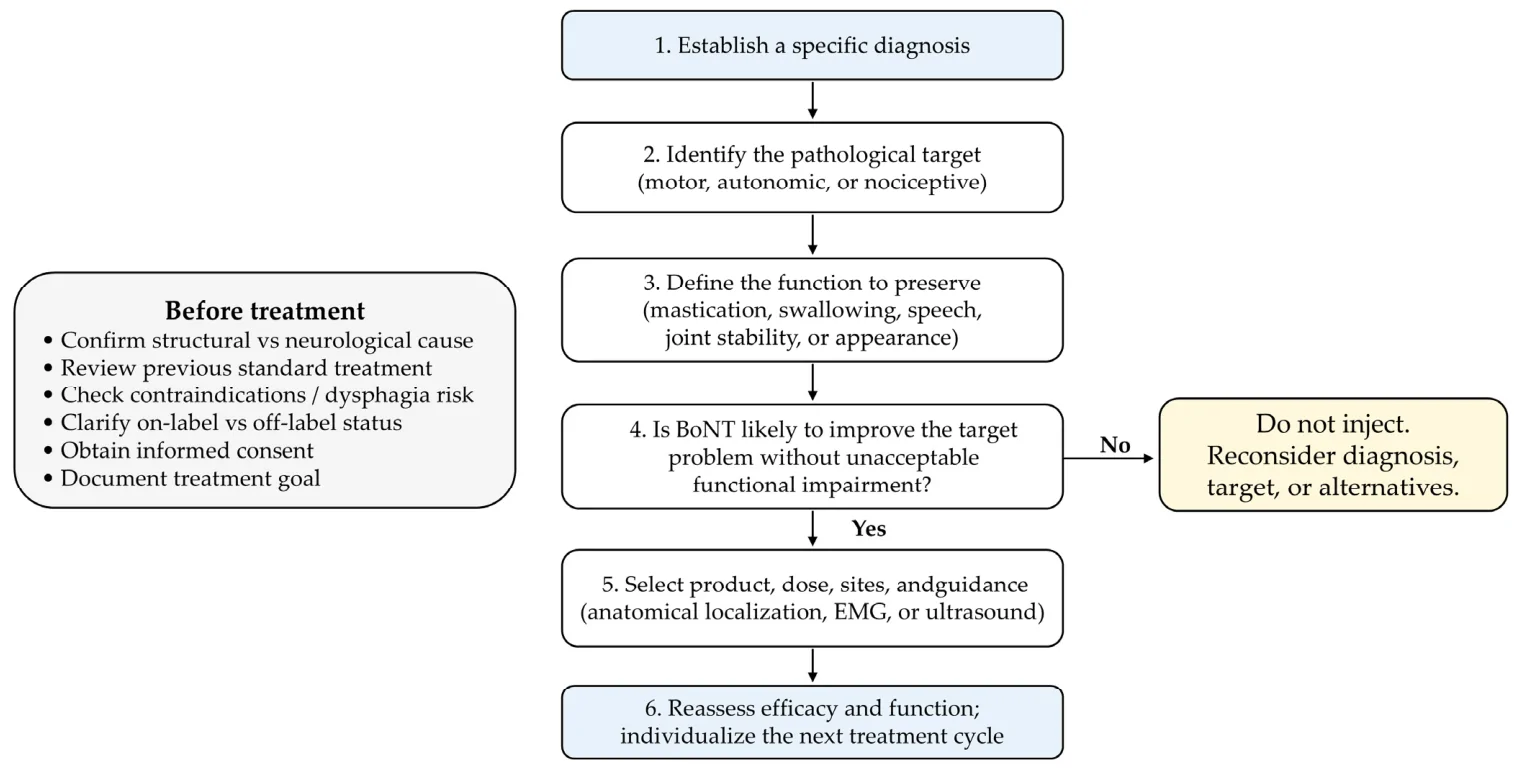
Diagnosis-target-function clinical decision pathway for BoNT therapy in the stomatognathic system. The pathway emphasizes diagnosis-driven rather than symptom-driven treatment, identification of the pathological target, preservation of essential oral functions, appropriate selection of product, dose, injection site, and guidance method, and reassessment of efficacy and adverse effects before subsequent treatment cycles.

ChatGPT (OpenAI, GPT-5.6 Sol; accessed September 2026) was used to assist in the development of Figure 1 and Figure 2 and the graphical abstract. GenAI was not used to generate, fabricate, collect, analyze, or interpret original study data. The author independently selected and verified the cited literature, checked all scientific and clinical statements against the relevant sources, reviewed and edited the generated material, and takes full responsibility for the accuracy, originality, and integrity of the final manuscript and figures.

## 3. Why the Stomatognathic System Is Uniquely Suited to Botulinum Toxin Therapy

### 3.1. Multiple Essential Functions Within a Confined Anatomical Region

Few anatomical regions participate simultaneously in as many complex functions as the stomatognathic system. Mastication requires precisely timed reciprocal activity of jaw-closing and jaw-opening muscles. Swallowing requires coordination of the tongue, suprahyoid muscles, pharyngeal structures, and palate. Speech depends on finely controlled movements of the tongue, lips, mandible, and soft palate, whereas facial expression and oral competence require coordinated activation of numerous small facial muscles.

Abnormal activity in any component can therefore produce disproportionate functional impairment. Hyperactivity of the masseter or temporalis may cause jaw-closing dystonia or contribute to painful masticatory muscle disorders. Inappropriate activation of the lateral pterygoid or suprahyoid muscles may cause dystonic jaw opening. Lingual dystonia may markedly impair speech and swallowing, while sialorrhea can affect communication, skin integrity, and social interaction.

Because BoNT provides localized, dose-dependent, and reversible modulation of neuromuscular or autonomic activity, it is particularly suited to disorders in which excessive or inappropriate activity can be attributed to identifiable anatomical structures.

### 3.2. Accessibility of Target Muscles and Glands

Many structures of the stomatognathic system are accessible by percutaneous or intraoral injection. Superficial muscles such as the masseter, temporalis, orbicularis oris, and selected facial muscles can often be identified clinically. Deeper structures, including the lateral and medial pterygoid muscles, lingual muscles, and selected suprahyoid muscles, can be approached with appropriate anatomical knowledge and electrophysiological or imaging guidance.

The major salivary glands are likewise accessible using ultrasound-guided techniques.

Accessibility, however, should not be equated with simplicity. The region contains major vessels, nerves, glands, the pharyngeal airway, and muscles essential for swallowing and articulation. Safe treatment therefore requires detailed anatomical knowledge.

### 3.3. Reversibility as an Advantage in a Multifunctional System

Permanent surgical weakening or resection of muscles involved in oral function may have irreversible functional consequences. By contrast, BoNT provides temporary chemodenervation, allowing the dose and target muscles to be individualized over successive treatment cycles.

This reversibility is particularly valuable when treating muscles that serve both pathological and physiological functions. For example, the goal of BoNT therapy for jaw-opening dystonia is not paralysis of all jaw-opening muscles, but selective reduction of pathological activity sufficient to restore coordinated mastication while preserving swallowing.

Similarly, masseter treatment must balance reduction of excessive force or muscle volume against preservation of adequate masticatory function.

### 3.4. Mechanistic Convergence Within One Anatomical System

The therapeutic breadth of BoNT in the stomatognathic system can be conceptualized along four partially overlapping axes: (1) neuromuscular chemodenervation for excessive or inappropriate muscle activity; (2) sensory and antinociceptive modulation for selected pain disorders; (3) autonomic cholinergic blockade for hypersecretory conditions; and (4) morphologic remodeling secondary to sustained reduction of muscle activity in aesthetic applications. A distinctive feature of the stomatognathic region is that all four mechanisms can be relevant in adjacent structures and, in some patients, simultaneously. The therapeutic objective should therefore be restoration or preservation of function with the lowest effective degree of biological modulation, rather than maximal paralysis.

## 4. Movement Disorders of the Stomatognathic System

Movement disorders constitute one of the best-established and most mechanistically direct applications of BoNT in the stomatognathic region.

### 4.1. Oromandibular Dystonia

OMD is characterized by sustained or intermittent involuntary contractions of the masticatory, lingual, and/or lower facial muscles. According to the predominant movement pattern, OMD can be classified as jaw-closing, jaw-opening, jaw-deviation, jaw-protrusion, lingual, lip, or mixed dystonia [10,11].

Clinical manifestations include impaired mastication, involuntary mouth opening or closing, tongue protrusion, abnormal lip movements, biting of the tongue or buccal mucosa, dysarthria, dysphagia, muscle pain, TMJ symptoms, and aesthetic disturbance.

BoNT is widely regarded as a first-line treatment for many patients with OMD, although regulatory approval varies across countries and formulations. A systematic review and meta-analysis of onabotulinumtoxinA demonstrated substantial overall clinical benefit while also highlighting the need for additional controlled studies and standardized outcome measures [11,23].

Target selection is phenotype dependent. Jaw-closing dystonia commonly requires treatment of the masseter and temporalis muscles, with the medial pterygoid added when necessary. Jaw-opening dystonia may involve the lateral pterygoid, anterior digastric, mylohyoid, and other suprahyoid muscles. Jaw-deviation and jaw-protrusion dystonia often require individualized treatment of the lateral pterygoid and other contributing muscles. Lingual dystonia is among the most technically demanding phenotypes because treatment must reduce pathological tongue activity without inducing excessive dysphagia or speech impairment [10,24,25].

Electromyographic guidance is particularly valuable for deep or functionally complex muscles because structural localization alone does not establish whether a muscle is actively generating the abnormal movement.

### 4.2. Task Specificity and Early Recognition

Task specificity is an important clinical feature of OMD. Abnormal movements may initially occur only during speaking, chewing, swallowing, singing, or another specific oral motor task.

This feature is highly relevant to dentistry. A patient whose mouth opens involuntarily only during mastication may simply report an inability to chew. Conventional examination may show adequate teeth, occlusal contacts, prostheses, masticatory muscle strength, and TMJ structure. If mastication itself is not observed, task-specific jaw-opening dystonia may remain undetected.

Such patients may undergo repeated occlusal adjustment, prosthesis replacement, splint therapy, or other dental procedures because their functional impairment is interpreted as structural rather than neurological.

Recognition of subtle task-specific abnormalities may therefore permit earlier diagnosis and treatment before disability becomes more extensive. For dental clinicians, this represents diagnostic stewardship: when the severity or task dependence of dysfunction is inconsistent with structural dental findings, the provoking task should be observed and a movement disorder considered before irreversible occlusal or prosthodontic intervention [10,45].

### 4.3. Oral and Orolingual Dyskinesia

Orofacial dyskinesias may involve repetitive or stereotyped movements of the jaw, lips, tongue, or facial muscles and are commonly associated with dopamine receptor-blocking drugs or neurodegenerative disorders.

BoNT may be useful when a limited group of muscles produces clinically significant symptoms, trauma, pain, or functional impairment. Treatment must be individualized because dyskinesias often involve complex multimuscle patterns and may respond less predictably than focal dystonia.

### 4.4. Bruxism

Bruxism is repetitive masticatory muscle activity characterized by clenching or grinding of the teeth and/or bracing or thrusting of the mandible. Contemporary consensus distinguishes sleep bruxism from awake bruxism and emphasizes that, in otherwise healthy individuals, neither behavior is necessarily a disorder in itself [27,28,29]. Treatment should therefore target clinically relevant consequences rather than the mere presence of bruxism.

BoNT has increasingly been used for bruxism associated with clinically meaningful consequences, particularly pain, muscle hypertrophy, dental complications, or failure of conservative management. Most protocols target the masseter, sometimes together with the temporalis. However, a 2025 overview of 14 systematic reviews found that, although many reviews reported reductions in pain, bruxism-related motor events, or bite force, methodological confidence was low or critically low; definitive conclusions therefore remain difficult [13,30].

Accordingly, BoNT should not be considered routine treatment for every patient with bruxism or treatment of the behavior itself. Patient selection should be based on clinically meaningful consequences, assessment and management of contributing sleep, medication-related, behavioral, or neurological factors, and a clearly defined treatment goal [13,27,28,31].

Repeated or excessive weakening of masticatory muscles also raises concerns regarding reduced bite force, muscle atrophy, altered mandibular biomechanics, and possible bone effects. Systematic reviews have identified signals of mandibular bone change after repeated injections into masticatory muscles, although the magnitude and long-term clinical significance in humans remain uncertain [46,47,48,49,50,51]. Optimal dose, retreatment interval, and long-term risk-benefit balance require further prospective study.

A distinct, still unproven, dental application is reduction of excessive occlusal loading in patients with load-related tooth or restorative complications or with reduced periodontal support. Traumatic occlusal forces can cause tooth wear, fracture, mobility, and injury to the periodontal attachment apparatus; however, current periodontal consensus does not support traumatic occlusal force as an initiator of periodontitis or periodontal attachment loss in humans, and evidence that it accelerates periodontitis remains limited [52,53,54,55]. Because BoNT/A can reduce maximal bite force in patients with bruxism, a plausible hypothesis is that carefully selected off-label treatment of jaw-closing muscles might reduce mechanical overload in patients who remain symptomatic despite appropriate periodontal and occlusal management [32,33]. This approach should not be presented as a treatment for periodontitis itself. Direct evidence that BoNT prevents tooth fracture, prosthetic failure, periodontal attachment loss, or tooth loss is lacking, and prospective studies are required before any periodontal-protective indication can be recommended.

### 4.5. Hemimasticatory Spasm

Hemimasticatory spasm is a rare movement disorder characterized by unilateral painful or painless contractions of the jaw-closing muscles, particularly the masseter and temporalis. It may be associated with localized scleroderma or facial hemiatrophy.

BoNT can markedly suppress involuntary contractions and is among the most effective symptomatic treatments. Accurate differentiation from OMD, hemifacial spasm, focal seizures, and TMD-related muscular symptoms is essential [34].

### 4.6. Tremor Involving the Stomatognathic System

Tremor may involve the jaw, tongue, soft palate, or other oral structures. Palatal tremor is particularly relevant to otological and oral symptoms and may cause rhythmic clicking, discomfort, or functional impairment [35].

BoNT may be considered in selected patients when the muscle responsible for the tremor can be identified. Because the therapeutic window is narrow in palatal and pharyngeal structures, dosing must be conservative to avoid velopharyngeal insufficiency, dysphagia, or speech disturbance.

### 4.7. Embouchure Dystonia

Embouchure dystonia is a highly task-specific movement disorder affecting wind instrumentalists. Abnormal activity may involve the lips, jaw, tongue, lower facial muscles, or combinations of these structures [36].

Because symptoms may occur only during instrument performance, routine neurological and dental examinations may be normal. Diagnosis therefore requires observation during the symptomatic musical task.

BoNT may benefit carefully selected patients when the principal abnormal muscle can be identified; however, excessive weakness can compromise the exceptionally fine motor control required for professional performance.

### 4.8. Functional Movement Disorders

Functional movement disorders can also involve the stomatognathic system [37]. Because a clinical response to BoNT does not establish organic dystonia, diagnosis should be based on positive features of functional movement disorders rather than exclusion alone.

BoNT may have a role in highly selected situations, but injection should not substitute for a positive phenomenological diagnosis. Treatment without diagnostic clarity may reinforce maladaptive illness behavior and expose patients to unnecessary functional weakness [10,37].

## 5. Pain Disorders

### 5.1. Myogenous Temporomandibular Disorders and Orofacial Myofascial Pain

Masticatory muscle pain is among the most common complaints in dental and orofacial pain practice. BoNT has increasingly been used for refractory myogenous TMD and chronic masticatory myofascial pain, but the evidence is heterogeneous and depends heavily on diagnostic phenotype, comparator, dose, and outcome measure [12,38,39,40,41].

BoNT has traditionally been assumed to relieve pain by reducing excessive muscle contraction and mechanical load. Analgesia, however, cannot be explained solely by muscle relaxation. Experimental and clinical evidence indicates that BoNT can inhibit nociceptive mediator release and modulate peripheral sensitization [7,8].

This distinction is clinically important because muscle activity and pain are not linearly related. Not all patients with masticatory muscle pain exhibit excessive contraction, and reducing muscle force does not necessarily abolish pain.

Accordingly, BoNT should generally be reserved for carefully selected patients with persistent symptoms after appropriate diagnosis and evidence-based conservative management. A 2024 umbrella review found moderate evidence for pain reduction versus placebo but no consistent superiority over standard treatments, whereas another 2024 systematic review and meta-analysis found no significant overall advantage over placebo for several TMD outcomes [12,40]. Current evidence therefore supports selective use in refractory cases rather than first-line or indiscriminate treatment.

### 5.2. Importance of Anatomical Subsite Selection

The masseter and temporalis are not functionally uniform muscles. Intramuscular nerve distribution, motor endplate zones, muscle architecture, tendon attachments, and nociceptive characteristics vary among subsites.

These differences suggest that injection location may be as important as total dose. Targeting regions rich in neuromuscular junctions may theoretically achieve efficient chemodenervation with lower doses, whereas painful regions may not correspond to areas of greatest motor activity. Motor-endplate-zone targeting in the masseter is therefore a biologically plausible, hypothesis-generating strategy rather than an established clinical standard [56,57,58,59,60,61,62,63,64,65].

Future studies should therefore move beyond asking whether BoNT should be injected into a given muscle and determine where within that muscle injection is most effective and safest.

### 5.3. Trigeminal Neuralgia

BoNT has emerged as a potential adjunctive treatment for trigeminal neuralgia, particularly in patients with an inadequate response to or intolerance of conventional pharmacotherapy [17,42].

Unlike treatment of muscular disorders, the presumed mechanism is primarily antinociceptive rather than paralytic. An updated 2024 systematic review and meta-analysis of 23 studies reported reductions in pain intensity and attack frequency and supported BoNT/A as a potentially effective and safe option for refractory trigeminal neuralgia, while emphasizing the need for additional high-quality studies [17].

This indication is particularly relevant to oral and maxillofacial practice because pain in the maxillary and mandibular divisions of the trigeminal nerve may present as dental pain. Misdiagnosis can lead to unnecessary endodontic treatment or tooth extraction.

Injection sites, doses, patient selection, and retreatment intervals require further standardization. BoNT should be positioned as an adjunct for selected refractory patients rather than a replacement for established pharmacological or neurosurgical treatment algorithms.

### 5.4. Other Chronic Orofacial Pain Conditions

BoNT has also been investigated for other neuropathic and chronic orofacial pain conditions. Interpretation remains difficult because diagnostic criteria and treatment protocols are heterogeneous.

A major future priority is precise phenotyping. Orofacial pain should not be treated as a single indication because neuropathic, nociplastic, muscular, articular, and dystonia-related pain may require substantially different therapeutic strategies.

## 6. Temporomandibular Joint Disorders

### 6.1. Recurrent Temporomandibular Joint Dislocation

Recurrent TMJ dislocation is a clear example of how muscle-directed BoNT treatment can modify an articular disorder.

The lateral pterygoid contributes to anterior translation of the mandibular condyle. Excessive or inappropriate activity may contribute to recurrent anterior dislocation in selected patients. BoNT injection into the lateral pterygoid can reduce pathological anterior traction and may decrease recurrence [18,19].

This approach may be particularly valuable in elderly or medically compromised patients for whom surgery carries increased risk and in patients whose dislocation is associated with neurological disease or dystonia. Evidence, however, remains at a low level: a historical series reported favorable outcomes in 21 patients [18], whereas a systematic review concluded that recurrent dislocation management, including BoNT, is still supported predominantly by case series and case reports [19].

Because the lateral pterygoid is a deep muscle adjacent to major vascular structures, accurate injection technique is essential. EMG can confirm activity in the target muscle, while ultrasound or other imaging modalities can assist anatomical localization when appropriate.

### 6.2. Relationship Between Dystonia and TMJ Dislocation

TMJ dislocation should not always be regarded as a primary joint disorder. In some patients, particularly those with severe jaw-opening OMD, recurrent or sustained involuntary opening may contribute secondarily to dislocation [10].

This distinction has important therapeutic implications. Treating the dislocated joint without controlling the underlying abnormal muscle activity may lead to recurrence. Conversely, chronic unreduced dislocation can itself cause severe structural dysfunction and mimic a persistent dystonic posture.

Neurological and structural causes should therefore be considered together.

### 6.3. Other TMJ-Related Indications

BoNT has been proposed for other TMJ symptoms, including clicking and internal derangement, but evidence for these applications remains limited.

Mechanical joint sounds can arise from disc displacement, joint morphology, ligamentous factors, or altered movement patterns and should not automatically be attributed to muscular hyperactivity.

BoNT should not be advocated for TMJ clicking alone without a convincing muscular mechanism; these applications should currently be regarded as investigational.

## 7. Salivary and Autonomic Disorders

### 7.1. Sialorrhea

Sialorrhea is one of the best-established non-muscular indications for BoNT in the orofacial region. It commonly occurs in neurological disorders such as Parkinson disease, amyotrophic lateral sclerosis, and cerebral palsy, in which impaired swallowing or oral motor control contributes to drooling. A systematic review and meta-analysis of 17 randomized studies involving 981 patients found moderate-certainty evidence that BoNT-A or BoNT-B reduces sialorrhea in adults, with most adverse effects being mild to moderate and self-limited [20].

Injection of BoNT into the parotid and/or submandibular glands reduces cholinergic stimulation of salivary secretion. Ultrasound guidance can improve anatomical localization, particularly for the submandibular glands and in patients with challenging anatomy [20].

Treatment may reduce drooling, aspiration-related complications, skin irritation, caregiver burden, and social embarrassment.

Excessive reduction of salivary flow, however, may cause xerostomia, thickened secretions, impaired oral clearance, dental caries, mucosal discomfort, or swallowing difficulty. Dentists are particularly well positioned to monitor these oral consequences.

### 7.2. Frey Syndrome

Frey syndrome results from aberrant autonomic reinnervation after parotid surgery or trauma and is characterized by gustatory sweating and flushing. Intradermal BoNT/A has shown durable symptomatic benefit in prospective series and is now a widely accepted minimally invasive treatment [21].

Frey syndrome illustrates a distinct mechanism of BoNT action in the maxillofacial region: inhibition of pathological cholinergic autonomic transmission rather than skeletal muscle relaxation.

### 7.3. Other Secretory Conditions

Potential applications to other localized secretory disorders continue to be explored. As with muscular treatment, accurate diagnosis and appropriate dosing are essential to avoid excessive physiological suppression.

## 8. Aesthetic Applications

### 8.1. Masseter Prominence

Treatment of masseter prominence or hypertrophy is one of the most widely recognized aesthetic applications of BoNT in East Asia and is increasingly used worldwide.

Repeated BoNT injection reduces masseter activity and subsequently muscle volume, producing narrowing of the lower face in appropriately selected patients.

An important regulatory development occurred in Japan in August 2026, when Botox Vista (onabotulinumtoxinA) received an additional indication for masseter prominence in adults younger than 65 years. Updated Japanese regulatory information and review documentation were issued on 24 August 2026 [16]. This approval represents a major milestone in the formal recognition of BoNT treatment targeting a principal masticatory muscle.

This development may have implications beyond aesthetic medicine. For the first time in Japan, a major masticatory muscle has become the target of a formally approved BoNT indication. The approval may increase clinician familiarity with masticatory muscle anatomy and BoNT injection and stimulate broader clinical and scientific interest in other stomatognathic applications.

Nevertheless, aesthetic and therapeutic treatment should not be conflated. Dose, treatment objective, injection distribution, patient selection, and outcome assessment differ between facial-contour reduction and treatment of dystonia, bruxism, or muscle pain.

### 8.2. Gummy Smile

Excessive gingival display during smiling may partly reflect hyperactivity of the upper-lip elevator muscles. A 2024 systematic review of 13 prospective controlled trials found consistent short-term reductions in gingival exposure, with maximal effects typically at 2–4 weeks and reported duration of approximately 12–24 weeks; commonly targeted muscles included the levator labii superioris alaeque nasi, levator labii superioris, and zygomaticus minor [22].

Excessive treatment, however, may cause smile asymmetry, lip incompetence, speech changes, or unwanted alteration of facial expression. Skeletal, dentoalveolar, periodontal, and muscular contributors should therefore be assessed, and injection pattern and dose individualized rather than applied uniformly [22].

### 8.3. Perioral Wrinkles and Lower Facial Aesthetics

BoNT is used for selected perioral and lower facial aesthetic indications, including dynamic wrinkles, downturn of the oral commissures, mentalis hyperactivity, and related conditions [44].

The functional importance of perioral musculature distinguishes this region from many upper-facial cosmetic injection sites. Even small overdoses can impair articulation, drinking, mastication, or oral competence.

Detailed understanding of oral function is therefore essential.

## 9. Why Dentistry and Oral and Maxillofacial Surgery Should Play a Greater Role

### 9.1. Anatomical Expertise

Dentists and oral and maxillofacial surgeons routinely evaluate the teeth, occlusion, masticatory muscles, TMJs, tongue, oral mucosa, salivary glands, and cranial nerves.

They also routinely perform local anesthetic injections, intraoral procedures, nerve blocks, and surgical approaches in anatomically complex regions.

These skills provide a strong anatomical foundation for BoNT treatment.

### 9.2. Functional Expertise

Dental clinicians assess functions that are rarely evaluated in comparable detail in other specialties, including mastication, occlusion, mandibular excursions, bite force, swallowing-related oral function, prosthetic stability, and interactions between dental structures and muscle activity.

This perspective is important because a BoNT injection that successfully reduces pathological contraction may still be clinically unsuccessful if it compromises mastication, swallowing, articulation, or prosthetic function.

### 9.3. Dentists May Be the First Clinicians to Encounter These Patients

Many patients with OMD, bruxism, masticatory muscle pain, trigeminal neuralgia, TMJ dislocation, or other stomatognathic disorders first consult dentists because their symptoms are perceived as dental, occlusal, prosthetic, or joint problems.

This creates both an opportunity and a risk.

The opportunity is earlier recognition of neurological or neuromuscular disease.

The risk is repeated irreversible dental treatment when the primary abnormality lies in motor control rather than dental structure.

Task-specific jaw-opening dystonia is a particularly illustrative example. Difficulty chewing may lead to repeated prosthodontic evaluations and adjustment or reconstruction of otherwise adequate occlusion. When involuntary jaw opening occurs only during mastication, routine examination may appear nearly normal; direct observation during eating can reveal the neurological mechanism.

Earlier recognition and appropriate referral may prevent unnecessary occlusal adjustment, repeated prosthesis fabrication, unnecessary splint therapy, and prolonged diagnostic delay. This is particularly relevant because patients with TMD-like symptoms may experience complex and lengthy diagnostic and treatment pathways [45].

Beyond avoiding ineffective treatment, early recognition may improve prognosis and quality of life when symptoms progress. In some patients, task-specific abnormalities initially limited to chewing may later involve speech or other oral functions. Severe jaw-opening dystonia may eventually interfere with mouth closure and contribute to recurrent TMJ dislocation; once dislocation becomes chronic and unreduced, surgical management under general anesthesia may be required. Such progression is not inevitable, and prospective longitudinal evidence is needed before a causal sequence can be established.

More broadly, recognition of early-stage movement disorders by dental specialists may reduce disability, secondary structural complications, diagnostic delay, repeated irreversible procedures, and healthcare utilization. Future studies should quantify these outcomes directly, including time to correct diagnosis, the number and cost of preceding dental procedures, patient-reported quality of life, and subsequent need for surgery.

## 10. The Risk of Overexpansion: BoNT Is Not Appropriate for Every Stomatognathic Symptom

The broad therapeutic potential described above should not be interpreted as justification for indiscriminate use of BoNT in dental practice.

Expansion without appropriate diagnosis could create a new category of iatrogenic problems.

Muscle pain does not necessarily indicate excessive muscle contraction. Bruxism does not automatically require muscle weakening. TMJ clicking is not necessarily caused by lateral pterygoid activity. Facial pain may be neuropathic, nociplastic, odontogenic, articular, muscular, or functional/psychogenic. Excessive reduction of salivary flow may also compromise oral health.

BoNT therapy should therefore be diagnosis-driven rather than symptom-driven. A practical framework is the diagnosis-target-function triad: establish the diagnosis, identify the anatomical and functional target responsible for the clinically relevant symptom, and define the function that must be preserved.

Figure 2 translates this triad into a practical clinical pathway. The key decision is not simply whether a symptom is potentially BoNT-responsive, but whether a specific pathological motor, autonomic, or nociceptive target can be identified and modulated while preserving the essential function of the same anatomical system.

### Representative Clinical Scenarios

Three brief scenarios illustrate the framework. First, a patient with apparently adequate dentition and prostheses who develops involuntary mouth opening only during chewing should be observed while eating; task-specific jaw-opening dystonia should be considered before further irreversible occlusal or prosthodontic treatment. Second, recurrent anterior TMJ dislocation associated with demonstrable excessive lateral pterygoid activity may be amenable to EMG-guided BoNT injection, whereas dislocation driven primarily by ligamentous laxity or structural abnormalities may require a different strategy. Third, paroxysmal electric-shock-like pain in a trigeminal distribution should prompt neurological diagnosis and standard pharmacological management before BoNT is considered as an adjunct for refractory trigeminal neuralgia. These examples emphasize that identical symptoms can arise from different mechanisms and therefore require different targets—or no BoNT treatment at all.

Before injection, clinicians should answer three fundamental questions: (1) What is the specific diagnosis? (2) Which anatomical structure is responsible for the clinically relevant symptom? (3) Is reducing the activity of that structure likely to improve the target problem without causing unacceptable functional impairment? The treatment objective and anticipated functional adverse effects should be documented before injection.

These principles are particularly important in the stomatognathic system because the same muscle may contribute simultaneously to mastication, swallowing, articulation, joint stabilization, and facial expression.

## 11. Precision Injection: From Anatomical to Functional Targeting

Future development of BoNT therapy should focus not only on selecting the correct muscle but also on identifying the correct functional target within that muscle.

### 11.1. Electromyography

Electromyography (EMG) provides information that anatomical imaging cannot: whether a muscle is active during the pathological movement.

This distinction is crucial in dystonia. A structurally normal muscle may be pathologically active, whereas an anatomically plausible muscle may not contribute to the abnormal movement.

EMG is particularly useful for deep muscles such as the lateral and medial pterygoids and for lingual muscles, in which inaccurate targeting can result in treatment failure or clinically important adverse effects.

### 11.2. Ultrasound

Ultrasound provides real-time visualization of muscle boundaries, glands, vessels, and adjacent structures. It is particularly useful for salivary gland injections and is increasingly used for masticatory muscle injections [66,67].

EMG and ultrasound should be regarded as complementary rather than competing modalities: EMG provides functional information, whereas ultrasound primarily provides structural information.

### 11.3. Motor Endplate Zone Targeting

BoNT acts predominantly at the neuromuscular junction. Injection near regions of dense motor-endplate distribution may therefore increase neuromuscular blockade per unit dose.

Motor-endplate-zone targeting is of particular interest in large muscles such as the masseter, where indiscriminate injection throughout the muscle may increase exposure of adjacent structures to toxin diffusion. Current anatomical evidence supports this as a rational strategy, but prospective clinical studies are required to determine whether endplate-zone targeting improves efficacy, permits dose reduction, or decreases adverse effects [56,57,58,59,60,61,62,63,64,65].

Anatomically optimized injection may ultimately allow lower doses, greater efficacy, fewer adverse events, and better preservation of physiological muscle function.

### 11.4. Digital and Three-Dimensional Guidance

Advances in cone-beam computed tomography, magnetic resonance imaging, intraoral scanning, CAD/CAM technology, and three-dimensional printing create opportunities for patient-specific injection planning.

Custom guides may help standardize injection trajectories for deep muscles, lingual targets, or other anatomically difficult sites and reduce operator dependence [68].

Such technologies may be particularly relevant to dentistry, where digital workflows and patient-specific guides are already routine in implantology and maxillofacial surgery.

## 12. Safety Considerations Specific to the Stomatognathic System

The principal advantage of BoNT—localized and reversible reduction of neuromuscular or autonomic activity—also underlies its principal risk. Major complications in the stomatognathic region include dysphagia, dysarthria, excessive masticatory weakness, unintended facial weakness, smile asymmetry, xerostomia, local hematoma, and spread to adjacent muscles [69,70].

### 12.1. Target-Specific Functional Risk and Dose Discipline

Risk varies substantially by target. Injection into superficial jaw-closing muscles is generally less likely to cause severe swallowing impairment than injection into lingual or suprahyoid muscles, whereas repeated high-dose masseter treatment may substantially reduce bite force. Lateral pterygoid injection requires awareness of adjacent vascular and neural structures, and lingual or suprahyoid injections require particular caution because small changes in muscle function may affect swallowing or articulation [10,24,25].

The principle of “start low and go slow” is therefore particularly applicable in this multifunctional region. Initial doses should generally be conservative and then individualized according to objective functional benefit and adverse effects.

### 12.2. Product-Specific Dosing, Dilution, and Immunogenicity

BoNT potency units are product-specific and should not be assumed to be interchangeable among formulations. Proposed conversion ratios are approximate and indication dependent [71,72]. Clinical reports should therefore specify the formulation, dilution, total dose, dose per site, number of injection sites, guidance method, and retreatment interval. Use of the lowest effective dose and an appropriate interval between treatments may also limit unnecessary exposure and reduce the risk of neutralizing-antibody-associated treatment failure [73,74].

### 12.3. Masticatory Muscle and Mandibular Bone Considerations

Repeated weakening of jaw-closing muscles may have consequences beyond transient reduction of bite force. Animal studies and human imaging studies have reported changes in masticatory muscle volume and mandibular bone parameters after repeated BoNT exposure, while recent systematic reviews identified a signal for bone effects but concluded that their magnitude and clinical relevance in humans remain uncertain [46,47,48,49,50,51]. This uncertainty supports conservative dosing, avoidance of unnecessary repeated high-dose treatment, and prospective monitoring of long-term function and morphology, particularly in younger patients and those receiving prolonged therapy.

### 12.4. Contraindications and Situations Requiring Particular Caution

Absolute contraindications are product-specific but generally include known hypersensitivity to the relevant BoNT preparation or its components and infection at the proposed injection site. Particular caution is warranted in patients with pre-existing dysphagia or respiratory compromise, peripheral motor neuropathies, amyotrophic lateral sclerosis, myasthenia gravis, Lambert-Eaton syndrome, or other disorders of neuromuscular transmission, because clinically significant weakness may be amplified. Concomitant aminoglycosides, curare-like agents, or other drugs that interfere with neuromuscular transmission may potentiate toxin effects. In the stomatognathic system, baseline masticatory weakness, severe xerostomia, aspiration risk, and dependence on a target muscle for speech or swallowing should also influence target and dose selection [43,69,70]. Pregnancy and lactation should be approached according to current product labeling and local guidance because evidence is limited.

Representative practical injection parameters are summarized in Table 2.

## 13. Levels of Evidence, Regulatory Status, and Ethical Considerations

A central challenge in stomatognathic BoNT therapy is the mismatch among widespread clinical use, heterogeneous evidence quality, and regulatory approval. Evidence is relatively mature for some neurological and autonomic indications, whereas several dental, pain, and TMJ applications rely on small trials, heterogeneous systematic reviews, or case series. Regulatory status also varies by country and product; approval of an indication for one formulation or jurisdiction should not be generalized automatically to another.

Off-label use is particularly common in stomatognathic practice. Regulatory approval is formulation-, indication-, age-, and jurisdiction-specific; approval of one BoNT product for a particular indication must not be extrapolated automatically to another product or clinical context. When off-label BoNT therapy is considered, the clinician should document the diagnosis, rationale for target selection, available evidence and its limitations, reasonable alternatives, anticipated duration of benefit, product-specific dose, and foreseeable functional risks. Explicit informed consent should distinguish established care from investigational or off-label practice. Commercial or convenience-driven expansion should be avoided, particularly when standard dental, periodontal, neurological, or pain treatments have not been appropriately considered. Product units are not interchangeable, and current labeling and local professional regulations should be checked before treatment [16,43,71,72].

Substantial clinical evidence exists for several neurological and autonomic indications. In contrast, some dental and TMJ applications are supported mainly by small observational studies, heterogeneous trials, or expert experience.

Evidence should therefore not be presented as equivalent across indications. For this narrative review, Table 1 uses pragmatic clinical position descriptors—relatively established, promising but heterogeneous, and emerging/investigational—rather than formal evidence grades. These descriptors integrate the maturity of the literature, consistency of clinical experience, and regulatory context, but should not be interpreted as GRADE recommendations.

For future clinical guidelines, indications may usefully be grouped into three categories:

Established or relatively well-supported applications

- selected forms of OMD;
- sialorrhea;
- Frey syndrome;
- selected aesthetic indications.

Promising applications with growing evidence

- refractory bruxism;
- masticatory muscle pain;
- trigeminal neuralgia;
- recurrent TMJ dislocation;
- hemimasticatory spasm.

Emerging or investigational applications

- selected TMJ internal derangements or joint sounds;
- selected functional movement disorders;
- highly task-specific conditions such as some forms of embouchure dystonia;
- other chronic orofacial pain syndromes without a clearly established mechanism.

Regulatory approval remains more limited than clinical use, and many applications are off-label despite longstanding clinical experience.

The recent Japanese approval for masseter prominence illustrates how regulatory status can change when an indication is supported by appropriately designed prospective clinical trials.

## 14. Future Directions

The next phase of BoNT therapy in the stomatognathic system should prioritize evidence, precision, and interdisciplinary collaboration rather than expansion of treatment volume alone.

### 14.1. Standardized Diagnostic Criteria

Clinical trials should enroll clearly defined phenotypes rather than broad categories such as “TMD,” “bruxism,” or “orofacial pain.”

### 14.2. Standardized Injection Protocols

Target muscles, anatomical sites, dose per site, total dose, dilution, injection guidance, and retreatment intervals should be reported explicitly.

### 14.3. Functional Outcome Measures

Reduction in muscle activity alone is an insufficient endpoint.

Studies should evaluate mastication, swallowing, speech, pain, oral function, patient-reported disability, quality of life, and treatment satisfaction.

### 14.4. Long-Term Safety

Repeated treatment requires longitudinal evaluation of muscle morphology, bite force, mandibular bone, joint loading, salivary function, swallowing, and other functional consequences.

### 14.5. Precision Treatment

The field is likely to move from muscle-based treatment toward patient-specific functional targeting that integrates clinical examination, EMG, ultrasound, imaging, three-dimensional anatomical data, and, where appropriate, digitally fabricated guidance systems.

### 14.6. Multidisciplinary Care

Neurologists contribute expertise in movement-disorder phenomenology and differential diagnosis; dentists and oral surgeons contribute expertise in occlusion, mastication, dental structure, TMJs, intraoral anatomy, and maxillofacial procedures. Otolaryngologists add expertise in swallowing, voice, and pharyngeal function, while pain specialists contribute knowledge of complex nociceptive mechanisms.

These disciplines should be complementary rather than competing.

The preferred future model is therefore neither “BoNT treatment by neurologists” nor “BoNT treatment by dentists,” but multidisciplinary diagnosis combined with treatment by clinicians who have the anatomical and functional expertise appropriate to each target.

### 14.7. Implementation, Diagnostic Delay, and Health Economics

Future studies should assess not only symptom reduction but also the consequences of earlier recognition and appropriate referral. Relevant outcomes include time from symptom onset to correct diagnosis, number of prior consultations and irreversible dental procedures, direct treatment costs, work or performance disability, quality-of-life impact, and avoidance of secondary complications or surgery. These outcomes may be particularly informative for task-specific OMD, trigeminal neuralgia presenting as dental pain, and other conditions that cross specialty boundaries.

For bruxism and other conditions associated with high occlusal loads, prospective studies should also determine whether BoNT-mediated bite-force reduction can reduce tooth fracture, restorative or implant complications, symptomatic secondary occlusal trauma, or deterioration of periodontally compromised teeth. Such outcomes should be studied only after adequate control of periodontal inflammation and should not be interpreted as evidence that BoNT treats periodontitis itself [52,53,54,55].

## 15. The Stomatognathic System as an Expanding Frontier

The therapeutic potential of BoNT in the stomatognathic system is notable not because any single indication is unique, but because several fundamentally different therapeutic mechanisms converge within one compact functional region (Figure 1; Table 1). BoNT can suppress involuntary movement or excessive muscle activity, modulate selected forms of peripheral pain, alter pathological muscular traction on the TMJ, reduce salivary or autonomic secretion, and reshape facial contour by reducing muscle activity and volume.

Several relevant disorders are also highly prevalent. Bruxism and TMD-related symptoms affect large segments of the population, while neurological disorders such as OMD are likely underdiagnosed in dental practice. Demand for lower-facial aesthetic treatment is increasing, and the recent regulatory approval of onabotulinumtoxinA for masseter prominence in Japan may further increase professional and public awareness.

At the same time, the target anatomy is familiar territory for dental and oral and maxillofacial clinicians.

These factors create an unusual convergence: a large population of potentially eligible patients, a broad spectrum of indications, identifiable and accessible anatomical targets, and a professional workforce already trained in the relevant anatomy and function.

The principal barrier is therefore no longer anatomical accessibility, but the need for accurate diagnosis, standardized treatment protocols, high-quality evidence, appropriate professional education, and suitable regulatory frameworks.

If these challenges are addressed, the stomatognathic system may evolve from a relatively underrecognized area of BoNT use into a major multidisciplinary therapeutic field.

## 16. Conclusions

The stomatognathic system represents an unusually broad yet incompletely explored field for BoNT therapy.

Its combination of motor, sensory, articular, secretory, and aesthetic functions creates potential indications ranging from OMD, dyskinesia, bruxism, tremor, and hemimasticatory spasm to masticatory muscle pain, trigeminal neuralgia, recurrent TMJ dislocation, sialorrhea, Frey syndrome, masseter prominence, and other aesthetic conditions.

Expansion, however, should be driven by accurate diagnosis and mechanistic understanding rather than nonspecific treatment of symptoms.

Dentists and oral and maxillofacial surgeons are well positioned to contribute because they possess detailed knowledge of stomatognathic anatomy and routinely assess mastication, occlusion, TMJs, oral function, salivary glands, and related structures. Collaboration with neurology, otolaryngology, pain medicine, rehabilitation medicine, and other specialties nevertheless remains essential.

The 2026 Japanese regulatory approval of onabotulinumtoxinA for masseter prominence represents both a symbolic and practical milestone, demonstrating that a principal masticatory muscle can become the target of a formally approved BoNT indication when supported by appropriate clinical development [16].

Future advances should focus on phenotype-specific diagnosis, precision targeting, optimized dosing, standardized outcomes, long-term safety, image- and EMG-guided injection, digital guidance technologies, and well-designed prospective multicenter studies.

With increasing recognition among dental professionals and continued advances in precision injection, the stomatognathic system has the potential to become a major expanding frontier for BoNT therapy.

## Figures and Tables

**Table 1:** Representative current and emerging applications of BoNT therapy in the stomatognathic system.

Therapeutic Domain	Disorder/Indication	Principal Target(s)	Main Therapeutic Rationale	Current Clinical Position
Movement disorders	Jaw-closing dystonia [10,11,23,24,25]	Masseter, temporalis, medial pterygoid	Suppression of excessive jaw-closing activity	Relatively well established
	Jaw-opening dystonia [10,11,23,24,25]	Lateral pterygoid, anterior digastric, other suprahyoid muscles	Reduction of inappropriate jaw-opening activity	Relatively well established in specialist practice
	Jaw-deviation/jaw-protrusion dystonia [10,11,23,24,25]	Lateral pterygoid ± other masticatory muscles	Correction of direction-specific dystonic activity	Specialist/individualized use
	Lingual dystonia [10,24,25,26]	Genioglossus and other intrinsic/extrinsic tongue muscles	Reduction of pathological tongue movements	Specialist use; narrow therapeutic window
	Oral/orolingual dyskinesia [10]	Symptom-producing masticatory, lingual, or perioral muscles	Reduction of focal disabling involuntary movements	Selected cases
	Bruxism [13,27,28,29,30,31,32,33]	Masseter ± temporalis	Reduction of clinically consequential excessive masticatory activity	Potential benefit; evidence quality remains limited
	Hemimasticatory spasm [34]	Masseter, temporalis ± medial pterygoid	Suppression of unilateral masticatory muscle spasms	Clinically useful despite rarity
	Jaw/tongue/palatal tremor [35]	Phenotype-specific muscles, including palatal muscles	Suppression of rhythmic involuntary activity	Selected cases
	Embouchure dystonia [36]	Lips, jaw, tongue, facial or masticatory muscles	Reduction of task-specific abnormal activity	Emerging/highly individualized
	Functional stomatognathic movement disorders [37]	Individually selected muscles	Symptomatic modulation in exceptional cases	Limited/controversial
Pain disorders	Myogenous TMD/masticatory myofascial pain [12,38,39,40,41]	Masseter, temporalis ± pterygoid muscles	Muscle relaxation and possible antinociceptive effects	Heterogeneous evidence; selected refractory cases
	Trigeminal neuralgia [17,42]	Painful trigeminal distribution; subcutaneous/intradermal or mucosal sites	Peripheral antinociceptive modulation	Growing evidence for refractory cases
	Other chronic orofacial pain [12,42]	Diagnosis-specific targets	Modulation of peripheral nociceptive signaling	Investigational/phenotype dependent
TMJ-related disorders	Recurrent TMJ dislocation [18,19]	Lateral pterygoid	Reduction of excessive anterior condylar traction	Promising but low-level evidence; selected patients
	Dystonia-associated TMJ dislocation [10,18]	Lateral pterygoid and other responsible dystonic muscles	Control of the underlying abnormal motor drive	Mechanistically rational; specialist use
	TMJ clicking/selected internal derangements [19,38]	Usually lateral pterygoid proposed	Modification of muscular contribution to joint mechanics	Investigational; evidence limited
Salivary/autonomic disorders	Sialorrhea [20,43]	Parotid and submandibular glands	Inhibition of cholinergic salivary secretion	Relatively well established; approved in selected jurisdictions
	Frey syndrome [21]	Intradermal treatment of affected skin	Inhibition of aberrant cholinergic sweating	Well established in clinical practice
Aesthetic/morphologic applications	Masseter prominence [14,15,16]	Masseter	Reduction of muscle activity and subsequent muscle volume	Established aesthetic application; approved in Japan for a defined adult indication in 2026
	Gummy smile [22]	Upper lip elevator muscles	Reduction of excessive upper-lip elevation	Established aesthetic use; effects are temporary
	Perioral wrinkles/lower facial hyperactivity [44]	Orbicularis oris, mentalis, depressor muscles, others	Reduction of dynamic muscle activity	Established aesthetic use; precise dosing required

Note: Current clinical position reflects overall clinical use and literature trends; evidence strength and regulatory approval vary by country, formulation, and indication. TMD, temporomandibular disorder; TMJ, temporomandibular joint.

**Table 2:** Representative practical parameters for BoNT injection in the stomatognathic system.

Indication	Target	Representative Starting Dose *	Reported/Typical Range *	Injection Sites	Guidance	Retreatment	Key Risks
Jaw-closing OMD	Masseter [10,11,23,26]	15–25 U/side; titrate	20–50 U/side	2–4 sites in lower/central muscle	Clinical ± US/EMG	~12–16 wk	Chewing weakness; smile asymmetry
Jaw-closing OMD	Temporalis [10,11,23,26]	10–15 U/side	10–30 U/side	2–4 sites	Clinical ± EMG	~12–16 wk	Temporal weakness; local discomfort
Jaw-opening/deviation OMD; recurrent TMJ dislocation	Lateral pterygoid [10,18,24,26]	10–20 U/side	10–40 U/side in specialist series	1–2 sites	EMG strongly recommended; US adjunct	~12–16 wk	Hematoma; dysphagia; excessive weakness
Jaw-closing OMD	Medial pterygoid [10,11,24]	10–15 U/side	10–25 U/side	1 site	EMG recommended	~12–16 wk	Dysphagia; deep-space bleeding
Lingual dystonia	Genioglossus/selected tongue muscles [10,11,25,26]	5–10 U/site	~10–30 U total, individualized	1–4 phenotype-specific sites	EMG recommended	~12–16 wk	Dysphagia; dysarthria; aspiration risk
Jaw-opening OMD	Anterior digastric/submental complex [10,11,23]	5–10 U/side	~5–20 U/side	1–2 sites	EMG ± US	~12–16 wk	Dysphagia; impaired jaw opening control
Bruxism/selected myogenous TMD	Masseter ± temporalis [12,13,30,32,33,38,39,40,41]	Masseter 20–30 U/side; temporalis 10–20 U/side	Trials vary widely; masseter 30–60 U/side has been studied	Masseter 3–5 sites; temporalis 2–4 sites	Clinical; US helpful	≥12 wk; often 3–4 mo	Reduced bite force; atrophy; possible bone effects
Chronic sialorrhea	Parotid + submandibular glands [20,43]	IncobotulinumtoxinA: 30 U/parotid + 20 U/submandibular per side	100 U total approved adult regimen	One site per gland/side	US preferred; landmarks acceptable per label	No sooner than 16 wk	Xerostomia; thick secretions; dysphagia
Frey syndrome	Affected intradermal area [21]	~1–2 U/cm^2^ (product/protocol dependent)	Titrate to Minor-test area	Grid-like intradermal injections	Anatomical mapping/Minor test	Often 6–12+ mo	Local facial weakness; diffusion
Gummy smile	LLSAN/LLS ± zygomaticus minor [22]	1.25–3 U/side or low-dose point-based protocols	Common reports up to ~7.5 U/side	1–2 points/side; phenotype dependent	Anatomical localization	~3–6 mo	Smile asymmetry; lip incompetence; speech change
Masseter prominence	Masseter [14,15,16]	Use current product-specific approved regimen where applicable	Product- and jurisdiction-specific	Label-defined multi-point masseter pattern	Anatomical ± US	Label/product-specific	Chewing weakness; asymmetry; contour change

* Doses are presented as representative published or specialist-practice values, primarily for onabotulinumtoxinA unless otherwise specified; they are not universal prescribing recommendations. Potency units are product-specific and are not interchangeable. Dilution is protocol- and product-dependent; a commonly reported onabotulinumtoxinA concentration is 2–5 U/0.1 mL after reconstitution with preservative-free saline, but clinicians should follow the relevant product label and local guidance. Most stomatognathic indications remain off-label in many jurisdictions. LLSAN, levator labii superioris alaeque nasi; LLS, levator labii superioris; OMD, oromandibular dystonia; US, ultrasound.

## Data Availability

No new data were created or analyzed in this study. Data sharing is not applicable to this article.

## Abbreviations

The following abbreviations are used in this manuscript:

BoNT	Botulinum neurotoxin
OMD	Oromandibular dystonia
TMJ	Temporomandibular joint
TMD	Temporomandibular disorder
EMG	Electromyography
